# A Rare Presentation of Isolated Right Colon Ischemia: The Mass-Forming Variant

**DOI:** 10.7759/cureus.13028

**Published:** 2021-01-31

**Authors:** M'hamed Turki, Anumita Chakraborti, Saif Bella, Amine Hila, Ali Timsar

**Affiliations:** 1 Internal Medicine, United Health Services, Johnson City, USA; 2 Gastroenterology and Hepatology, State University of New York (SUNY) Upstate Medical University, Syracuse, USA; 3 Gastroenterology and Hepatology, United Health Services, Johnson City, USA; 4 Pathology, United Health Services, Johnson City, USA

**Keywords:** ischemic colitis, mass-like lesions in colon, right sided colon ischemia

## Abstract

Ischemic colitis (IC), the most common gastrointestinal ischemia, remains an enigmatic disease with a wide array of pathogenic mechanisms and injuries along with variable outcomes. Among this group, isolated right colon ischemia (IRCI) appears to be a distinct entity, with its own pathophysiology, clinical presentation, and higher morbidity and mortality compared to left-sided colitis. IRCI is the most common site of mass-forming ischemic colitis. Colonoscopy with biopsy remains the key to diagnosis for this former entity. IRCI management is the same as for other IC and complete resolution of the mass is expected within weeks.

## Introduction

Ischemic colitis (IC), the most common gastrointestinal ischemia, remains an enigmatic disease with a wide array of pathogenic mechanisms and variable outcomes [1]. Compared to left-sided colitis, isolated right colon ischemia (IRCI) has higher morbidity and mortality, it is also the most common site of mass-forming ischemic colitis [2,3]. Here, we report two cases of IRCI that present as a pseudotumor and emphasize the singularity and implications of this variant.

## Case presentation

Case 1

**Figure 1:**
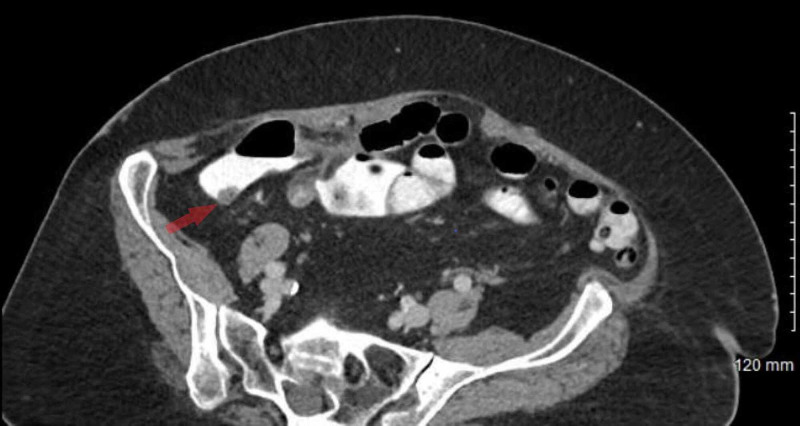
Axial view of a CT of the abdomen and pelvis with oral and intravenous contrast Mass-like thickening of the proximal right colon near the ileocaecal valve, measuring 6.3 cm in length, involving the proximal aspect of the ascending colon and the adjacent cecum with adjacent fat stranding (Transparent red arrow).

An 82-year-old Caucasian female with a history of hypertension, gastroesophageal reflux disease, dyslipidemia and gout, presented with right lower quadrant (RLQ) abdominal pain followed by a loose non-bloody bowel movement. Her symptoms were associated with tiredness, nausea, subjective fever and chills. She was hemodynamically stable on presentation. Physical examination revealed RLQ fullness to palpation. Initial blood work was remarkable for white blood cell count (WBC) 20.9 x 10^9^/mm^3^, hemoglobin 11.8 g/dl and blood urea nitrogen (BUN) 25 mg/dl. Computed tomography (CT) of the abdomen and pelvis showed a 6.3 cm mass-like thickening of the proximal right colon near the ileocecal valve which was concerning for colonic neoplasm (Figure 1).

**Figure 2:**
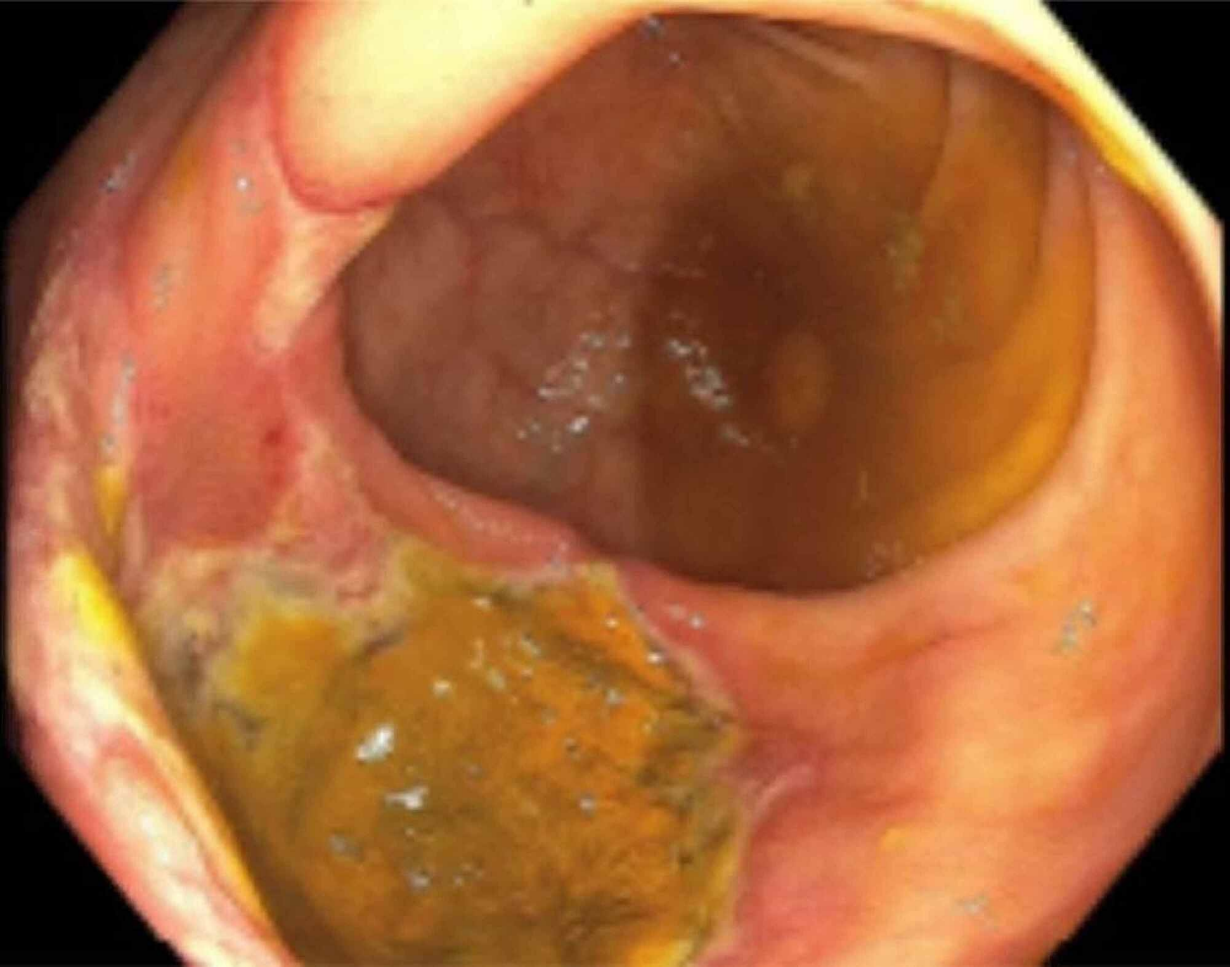
Colonoscopy image, at the level of the cecum A sample of the multiple 20 mm ulcers found in the cecum and ascending colon.

Carcinoembryonic antigen was within normal limits. Stool studies were negative for enteric pathogens or Clostridium difficile infection and fecal calprotectin level was elevated at 309 mcg/g. Colonoscopy demonstrated an area of moderately congested and ulcerated mucosa in the ascending colon and cecum suspicious for ischemic colitis (Figure 2).

Biopsies showed ulceration and granulation tissue and ruled out dysplasia or malignancy. The patient was treated successfully with intravenous (IV) fluids, bowel rest regimen and IV antibiotics.

Case 2

**Figure 3:**
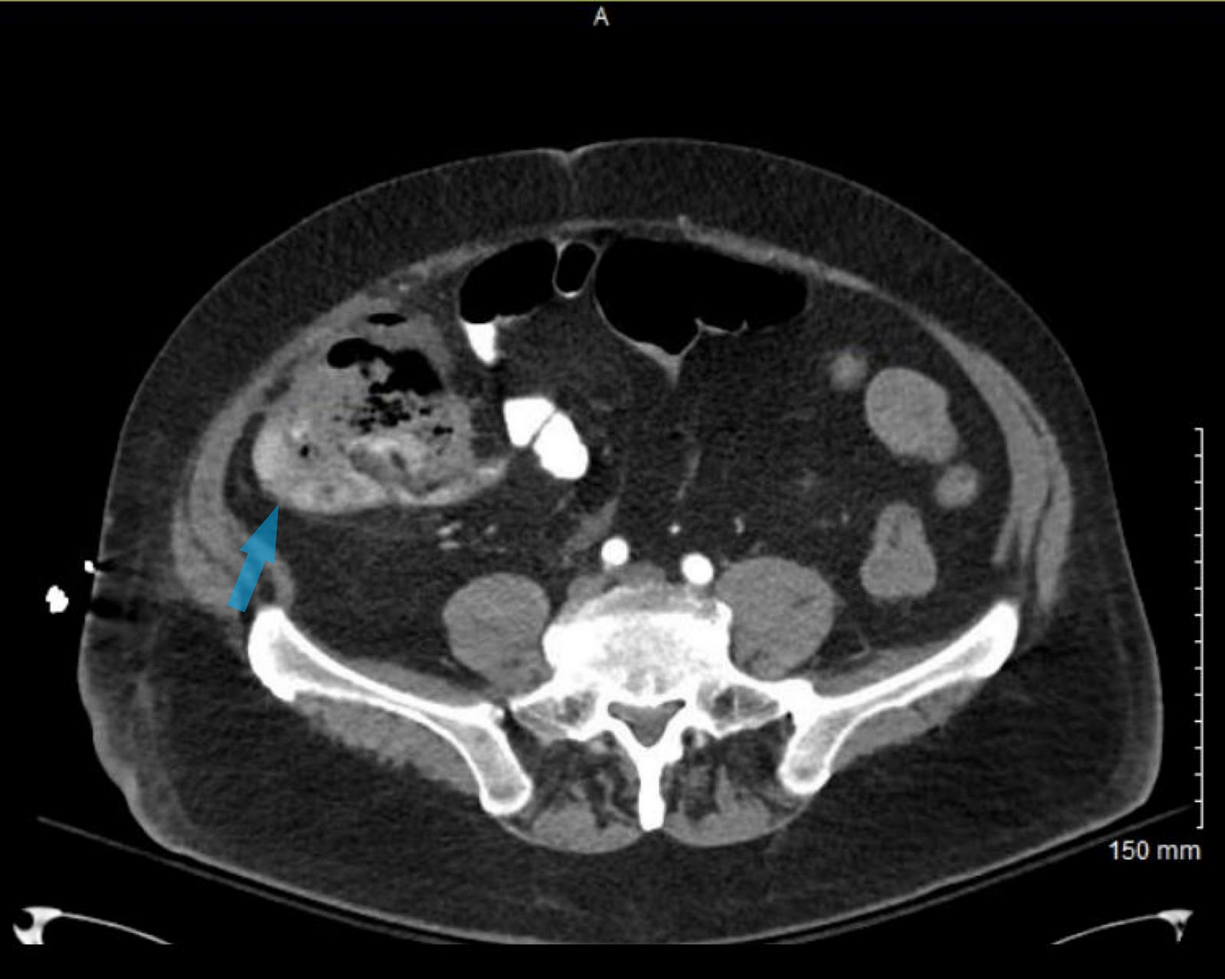
Axial view of a CT of the abdomen and pelvis with oral and intravenous contrast An 8-cm cecal mass (Transparent blue arrow) with loss of the fat plane between the cecal mass and the anterior lateral right abdominal wall with possible invasion of the wall.

A 70-year-old male with a pertinent history of coronary artery disease (CAD), hypertension, prediabetes and hypothyroidism underwent an off-pump coronary artery bypass graft. Ten days later, the patient presented to the hospital complaining of sharp colicky RLQ abdominal pain and dark stools. Blood pressure was down to 80/40 and improved with intravenous fluid resuscitation. Physical examination revealed RLQ tenderness. Blood workup was remarkable for sodium 134 mmol/L, BUN 25 mg/dl, lactate dehydrogenase (LDH) 673 U/L, WBC 18.9 x 10^9^/L and hemoglobin 9 g/dl. CT angiography of the abdomen and pelvis disclosed a 6-cm cecal mass concerning for colon cancer, high-grade stenosis of the proximal celiac artery and mild stenosis of the proximal mesenteric artery (Figure 3).

**Figure 4:**
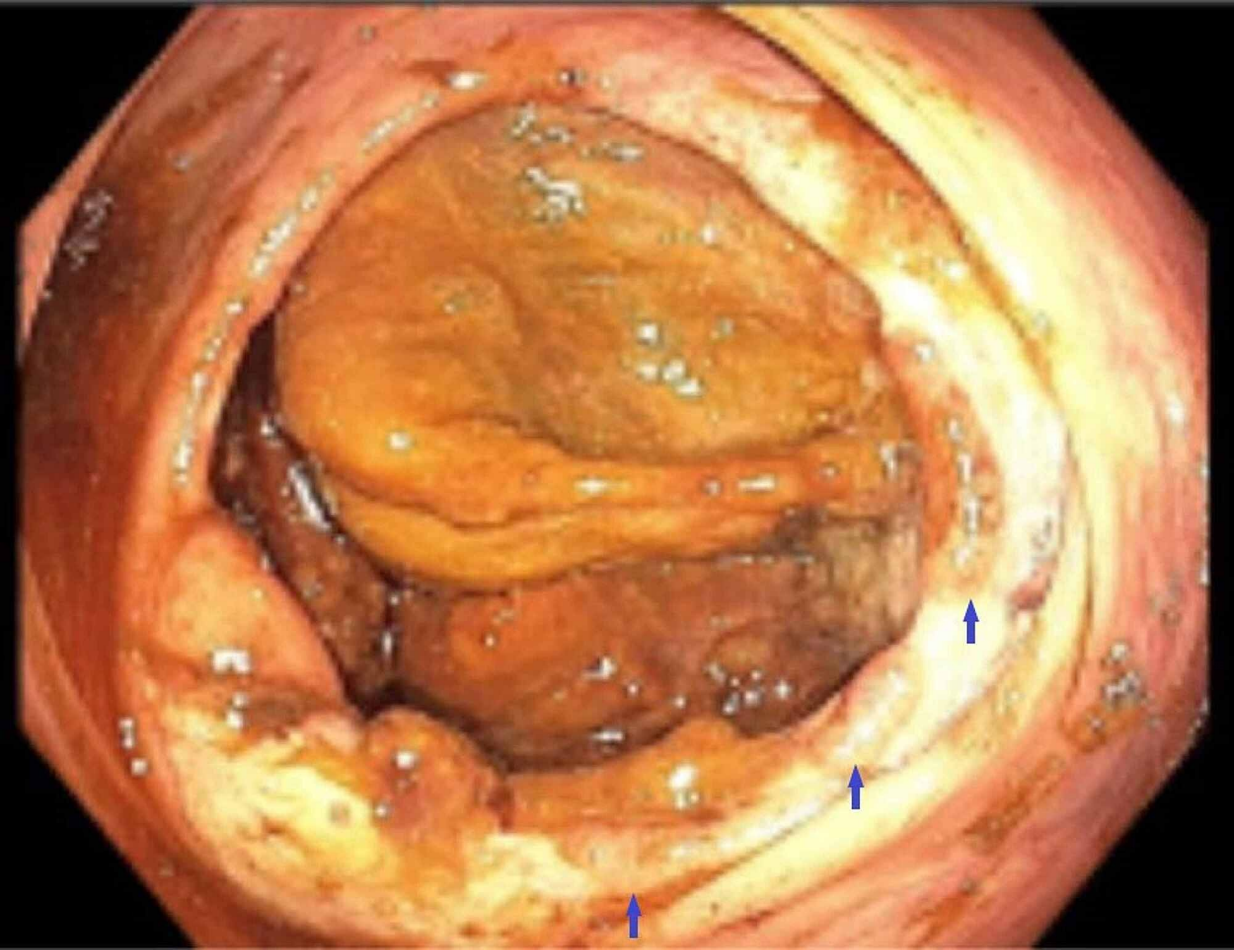
A colonoscopy image, at the level of the cecum Multiple cecal ulcers (Blue arrows).

Colonoscopy exhibited multiple ulcers in the cecum and at the ileocecal valve (Figure 4).

Biopsies demonstrated focally ulcerated colonic mucosa with fibrinopurulent exudate highly suspicious for ischemic colitis. The patient improved on intravenous fluids and antibiotics. Repeat colonoscopy eight months later showed complete resolution of the colitis.

## Discussion

IC is a nonocclusive disease resulting from a low flow state in the microvasculature of the colon. It represents the most common form of gastrointestinal ischemia, accounting for 50 to 60% of all ischemic events [1].

Typical patients with IC are not severely ill and present with vague symptoms (abdominal pain, the urge to defecate, maroon or bright red blood per rectum) that could be easily mistaken for infectious colitis or inflammatory bowel disease. The most common nonvascular CT findings in IC are segmental colonic wall thickening and pericolonic fat stranding [4]; these remain rather nonspecific signs [5]. Thus, early colonoscopy under minimal insufflation with biopsy remains the test of choice; findings can range from edema and erythema to pseudotumor or even gangrene. Serology (hemoglobin, WBC, BUN, LDH and sodium) has no diagnostic role and is only useful for risk stratification [6]. Overall, IC is a self-limited disease, as most cases resolve spontaneously and do not require specific therapy.

Over the last two decades, IRCI has been identified as a separate subgroup. IRCI arouses many questions most of which remain unanswered. The ‘watershed areas’ in the left colon have been historically thought to be the sites of poor perfusion making the right colon an unexpected area of primary ischemia. It has also been noticed that patients with IRCI usually carry a higher rate of comorbidities including CAD, atrial fibrillation and dialysis dependency [7]. Clinically, these patients present with a predominance of pain over rectal bleeding which is consistent with our two cases. In fact, the American College of Gastroenterology (ACG) guidelines strongly recommend considering the diagnosis of non-IRCI in patients with hematochezia. Compared with all other colon segments ischemia, IRCI has worse outcomes, a higher need for surgery, and a greater 30-day mortality rate [2,3]. Due to these specificities, IRCI pathophysiology has been hypothesized to be different from other IC. It is thought that IRCI could be secondary to a large vessel disease and could, in fact, herald, acute mesenteric ischemia. Longstreth and Hye [3] found in their retrospective study including 49 patients with IRCI that 11.4% of them were suffering from symptomatic large visceral artery occlusion causing abdominal angina. Cardiac arrhythmia and malignancies were also frequently diagnosed during the follow-up of these patients. Therefore, CT angiography is strongly recommended in patients with IRCI and, if not conclusive, a traditional splanchnic angiography should be considered. Note that CT with IV contrast is not appropriately timed for arterial visualization and is not sufficient in this setting.

As per ACG classification for IC, the presence of IRCI on imaging or colonoscopy represents alone a severe disease regardless of clinical presentation and serology: patients should be transferred to an intensive care unit, initiated on supportive treatment and broad-spectrum antibiotics and an emergent surgical consultation must be ordered [6].

IRCI is also famous for being the area of predilection for a rare variant called ‘mass-forming’ ischemic colitis (MFIC) by Khor et al. [8] and present in both of our patients. This IC mimics a tumor on CT or colonoscopy. It is thus crucial to have a high index of suspicion in recognizing this self-limited entity to prevent unnecessary surgery. According to some authors, this pseudotumor appearance could be due to submucosal edema and hemorrhage [1,9]. Colonoscopy with biopsy is the key to diagnosis. The management is the same for IRCI and complete resolution of the mass is expected within weeks.

## Conclusions

IRCI is a rare clinical finding with a poorly understood pathophysiology. Nevertheless, it is accepted today that IRCI is the disease of vasculopath patients. The MFIC belongs almost exclusively to the IRCI and remains the subject of multiple controversies. In the clinical setting, the provider should be aware of this rare presentation of IC and of its unfavorable outcome. No specific treatment is available for the management of IRCI and MFIC, and supportive measures in an intensive care unit remain the rule.
